# Aurora B inhibitors promote RB hypophosphorylation and senescence independent of p53-dependent CDK2/4 inhibition

**DOI:** 10.1038/s41419-024-07204-5

**Published:** 2024-11-09

**Authors:** Shivam Vora, Ariel Andrew, Ramyashree Prasanna Kumar, Deborah Nazareth, Alexis Bonfim-Melo, Yoon Lim, Xin Yee Ong, Madushan Fernando, Yaowu He, John D. Hooper, Nigel AJ McMillan, Jelena Urosevic, Jon Travers, Jamal Saeh, Sharad Kumar, Mathew JK Jones, Brian Gabrielli

**Affiliations:** 1 Mater Research Institute, The University of Queensland, Brisbane, QLD Australia; 2 Frazer Institute, The University of Queensland, Brisbane, QLD Australia; 3 Centre for Cancer Biology, University of South Australia, Adelaide, SA Australia; 4 Menzies Health Institute Queensland and School of Medical Science, Griffith University, Gold Coast, QLD Australia; 5 Bioscience, Research and Early Development, Oncology R&D, AstraZeneca, Cambridge, UK

**Keywords:** Senescence, Targeted therapies

## Abstract

Aurora B kinase (AURKB) inhibitors have been trialled in a range of different tumour types but are not approved for any indication. Expression of the human papilloma virus (HPV) oncogenes and loss of retinoblastoma (RB) protein function has been reported to increase sensitivity to AURKB inhibitors but the mechanism of their contribution to sensitivity is poorly understood. Two commonly reported outcomes of AURKB inhibition are polyploidy and senescence, although their relationship is unclear. Here we have investigated the major cellular targets of the HPV E6 and E7, p53 and RB, to determine their contribution to AURKB inhibitor induced polyploidy and senescence. We demonstrate that polyploidy is a universal feature of AURKB inhibitor treatment in all cell types including normal primary cells, but the subsequent outcomes are controlled by RB and p53. We demonstrate that p53 by regulating p21 expression is required for an initial cell cycle arrest by inhibiting both CDK2 and CDK4 activity, but this arrest is only triggered after cells have undergone two failed mitosis and cytokinesis. However, cells can enter senescence in the absence of p53. RB is essential for AURKB inhibitor-induced senescence. AURKB inhibitor induces rapid hypophosphorylation of RB independent of inhibition of CDK2 or CDK4 kinases and p53. This work demonstrates that p53 activation determines the timing of senescence onset, but RB is indispensable for senescence.

## Introduction

Aurora kinase inhibitors (AURKi) have been investigated in a broad range of cancers, but as yet none have been approved for clinical use [1]. Many of the developed AURKi have activity towards all three Aurora kinases, although AURKA and AURKB are the primary targets in cancers [2]. AURKA and AURKB are functionally distinct regulators of progression through mitosis. AURKA is essential for centrosome maturation and regulates mitotic entry, AURKB regulates exit from mitosis and controls correct partitioning of the replicated genome [3]. Selective AURKA inhibition causes defects in centrosome separation and mitotic spindle formation, resulting in spindle assembly checkpoint (SAC)-dependent arrest, mitotic slippage and apoptosis [4–6]. Inhibition of AURKB disrupts normal chromosome alignment and segregation during mitosis, premature inactivation of SAC-dependent mitotic arrest and cytokinesis failure resulting in tetraploidy after the first failed cytokinesis [4–6]. This tetraploidy triggers a p53-dependent cell cycle arrest through HIPPO pathway activation to block endoreplication [7–9], although the existence of this checkpoint arrest has been controversial [10, 11].

The outcomes of the mitotic defects caused by AURKi is commonly apoptosis for the AURKA selective inhibitors, and polyploidy, senescence or apoptosis for the AURKB selective and dual inhibitors [4, 6, 12–17]. However, the molecular features that define whether AURKB inhibitors (AURKBi) trigger these outcomes have not been defined. For example, the ability of AURKB inhibition to promote senescence appeared to be cell line dependent [18–21]. Key molecular determinants of senescence are RB and p53 tumour suppressor [22], however their contribution to AURKBi induced senescence has not been defined. P53 has been implicated in the cell cycle arrest after AURKB inhibition as a consequence of tetraploidy checkpoint activation [8], and loss of p53 increases the proportion of polyploid cells after AURKBi treatment [5]. The role of p53 is to increase the expression of the CDK inhibitor p21WAF1 that inhibits CDK2-cyclin which is responsible for the G1 phase cell cycle checkpoint arrest [8]. CDK2 inhibition also drives the DREAM repressor complex to downregulate expression of a large number of cell cycle regulators thereby inhibiting cell cycle progression and promoting senescence [23–25]. However, the contribution of p53 to AURK inhibitor-induced senescence has been questioned [26]. RB is also implicated in senescence through inhibition of E2F transcriptional activity through CDK4-Cyclin D/p16INK4A-dependent regulation of RB activity [22, 27]. The RB-E2F complex represses E2F regulated gene expression, although there is a strong overlap between RB-E2F and DREAM complex repressed genes [23]. AURKB has been reported to phosphorylate one of the 14 CDK-dependent phosphorylation sites on RB [28], although the effect of the phosphorylation of this single site on RB has modest effects on RB function [29] suggesting that loss of this single phosphorylation is unlikely to be responsible for AURKBi-induced senescence. Here we have investigated the mechanism of AURKBi-induced senescence and the contribution of RB and p53 pathways to this senescence.

## Materials and methods

### Cell lines and culture conditions

HCT116 (colorectal carcinoma) HeLa, CaSki and C33A cell lines were obtained from American Type Culture Collection (Manassas, VA, USA). The HT1080 (fibrosarcoma) cell line was purchased from CellBank Australia (Westmead, NSW, Australia). H322, H358 (non-small cell lung cancer) and H69, H2141 (small cell lung cancer) were provided by Dr Jill Larsen (QIMRB). Parental HCT116 and HCT116 p53^−/−^ [30] were used to produce the respective RB^−/−^ lines using CRISPR-Cas9 deletion. Parental MCF7 and RB^−/−^ derivative generated using CRISPR-Cas9 deletion technology (Synthego, Redwood CA, USA). The cell lines were cultured as described previously [14]. Human cervical keratinocytes (HCK) stably expressing TERT [31] were provided by Professor Nigel McMillan from Griffith University (Gold Coast, QLD, Australia) and cultured in Keratinocyte Serum Free Medium (GIBCO) supplemented with Bovine Pituitary Extract (50 μg/ml) and Epidermal Growth Factor (5 ng/ml) as well as 0.035 mM of Pen/Strep and CaCl2. The cell cultures were maintained at 37 °C, in low oxygen (2% O_2_ and 5% CO_2_).

### Lentiviral transduction

The HCT116 E7, CDK4R24C cell lines and HCK E6, E7 lines were generated by lentiviral transduction [30]. The HPV16 E7 inserts were synthesis with a with V5 Tag and flanking attB sites for Gateway cloning. HCT116 wild-type, p53^−/−^ and p21^−/−^ were transduced to express a CDK2 and CDK4 activity biosensor system [31].

### Live-cell imaging

HT1080 and HCT116 cells were seeded in a 12-well plate in triplicates and treated with DMSO (control) and AURKi. Live cell imaging was performed and manual analysis was as described previously [14].

### Flow cytometry

Cells were harvested and fixed as described previously [14]. The FACS analysis was conducted using the Beckman Coulter CytoFLEX-S. DNA content (PI signal) was analysed ungated, using the PE filter. Data was analysed on Flowjo (version 7.6.4, Becton, Dickinson & Company).

### Immunoblotting and immunoprecipitation

For immunoblotting, cell pellets were lysed in NETN lysis buffer as described previously [14]. The blots were probed for proteins overnight, using primary antibodies against V5 tag (Abcam), p107, Cyclin D1, Cyclin E1, CDK4, RRM2, Cyclin A2, Cyclin B1 (Cell Signaling), RB, pRB S807/811, pRBS780, Top2A, pNPM1 T199, p27, Survivin, pPP1 T320, pH3 S10 and histone H3 (Cell Signaling Technology), p130, hypophosphorylated RB, p21, p16, AURKA, AURKB (Becton Dickinson) and p15, α-tubulin (Sigma Aldrich). Proteins were visualized using chemiluminescence detection. The data are from two technical replicates from at least two biological replicates.

### Senescence assay

Senescent cells were stained for senescence associated β-galactosidase (SA-β-gal) using 5bromo-4-chloro-3-indoyl β-galactosidase (X-gal). Cell lines were seeded in a 12-well plate in triplicates, treated for 48 h, then washed to remove drugs and fresh media applied for a further four days. The cells were partially fixed with 4% PFA for 3 min then incubated with X-gal solution overnight at 37 °C. All images were taken at 10× magnification on an Olympus IX73 inverted microscope (bright- field). The number of senescent cells were counted, and data analysed in GraphPad Prism using a 2-way ANOVA with Tukey’s range test. Data was presented as mean ± standard deviation (SD).

### High content imaging

Cells were grown in 96 well plates and labelled with EdU for 2 h prior to fixing with 4% PFA. Cells were then stained for EdU, p53 (Santa Cruz DO-1), Ki67 (Dako) then imaged and analysed. Cells expressing the CDK2 and CDK4 reporter system were grown in 96 well plates, and after treatment, fixed and stained with DAPI. Cells were imaged using InCell 6500HS imager and analysed using Cell Profiler software [32], and data processed using R Studio [33]. Statistical analysis was performed using 2way ANOVA multiple comparisons tests for the HCT116 cell line panel, or as described in the figure legends.

## Results

### Loss of RB and p53 function bypass AURKBi-induced cell cycle arrest

**Fig. 1:**
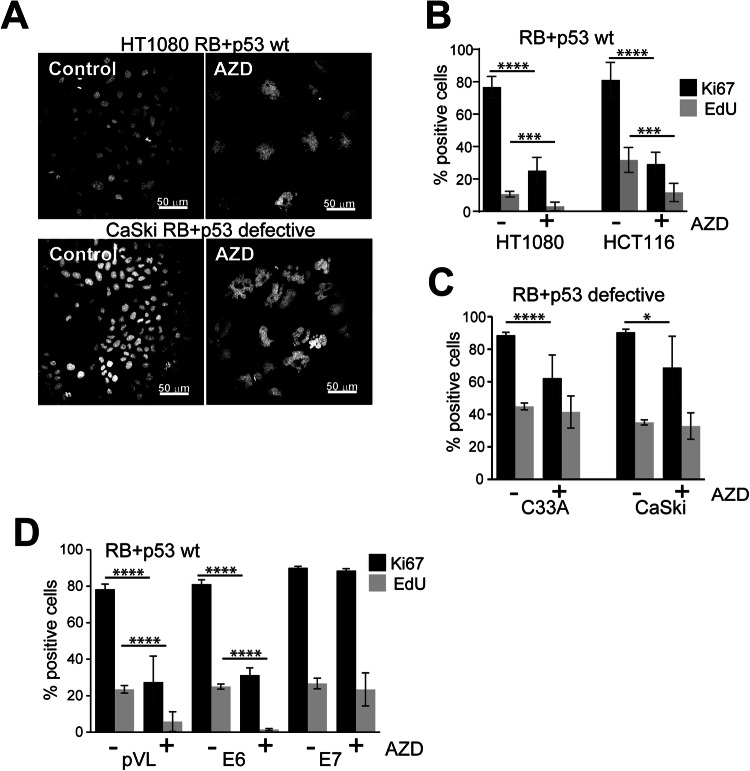
Loss of RB and p53 bypasses AURKBi-induced cell cycle arrest. **A** RB+p53 wild type HT1080 and RB+p53 defective CaSki cells were treated for 3 days with and without (control) 0.2 μM AZD2811 (AZD; AURKBi) then fixed and stained for DNA. Bars = 50 μm. **B** RB+p53 wild type HT1080 and HCT116 cells, **C** RB+p53 defective C33A and CaSki cells, and **D** immortalised human cervical keratinocytes expressing either empty vector (pLV), HPV E6 or E7 oncogenes, were treated for 6 days with 0.2 μM AZD2811, labelled for 2 h with EdU then stained for Ki67 and EdU incorporation. Cells were imaged using high content imaging and >1500 cells/well from at least three replicate wells were analysed. The percent positive staining is shown. Mean ± SD are shown. Data was collected from at least two independent experiments. Nonparametric t-test were used to compare control and treated data. The absence of comparison indicates no significant difference (***p > 0.001, ****p > 0.0001).

The role of RB and p53 in the cell cycle arrest and senescence in responses to AURKB inhibition was investigated in RB+p53 wild type and RB+p53-defective cells using the AURKB-selective inhibitor AZD2811. A universal outcome of AURKB inhibition was failed anaphase and cytokinesis resulting in polyploid, indicated by the increase size of the multilobed and fractured nuclei and increased DNA content, and confirmed using another AURKB selective inhibitor BI831266 [34] and a potent pan-AURK inhibitor AMG900 [35] (Figs. 1A and S1A). AZD2811 treatment resulted in the loss of proliferative markers Ki67 and EdU in RB+p53 wild type HCT116 and HT1080 cells (Fig. 1B), and while there was significant reduction in Ki67 staining there was no effect on EdU incorporation in RB+p53 defective cervical cancer lines (Fig. 1C). The same pattern was observed with the other AURKB inhibitors (Fig. S1B). The outcome of the extended cell cycle arrest in the RB+p53 wild type cell lines was senescence, evidenced by the increased senescence marker senescence associated β-galactosidase (SA-β–Gal) and senescence associated secretory phenotype factors [36] (Fig. S2A, B).

The role of RB and p53 in AURKBi-mediated cell cycle arrest was also demonstrated using immortalised human cervical keratinocytes (HCK) [37] transduced with lentivirus expressing either HPV16 E6 or E7 oncogenes that effectively ablated p53 or RB, respectively (Fig. S2C; loss of p107 is an indicator of RB disruption [38]). Additionally, HPV16 E7 is known to disrupt the DREAM complex function directly [24, 39]. HPV E6 had little effect on loss of proliferative markers with AZD2811 treatment, whereas HPV E7 expression permitted continued expression of these markers, suggesting RB was critical for AZD2811-induced cell cycle arrest (Fig. 1D).

### Cells must fail cytokinesis twice before p53-dependent cell cycle arrest is imposed

**Fig. 2:**
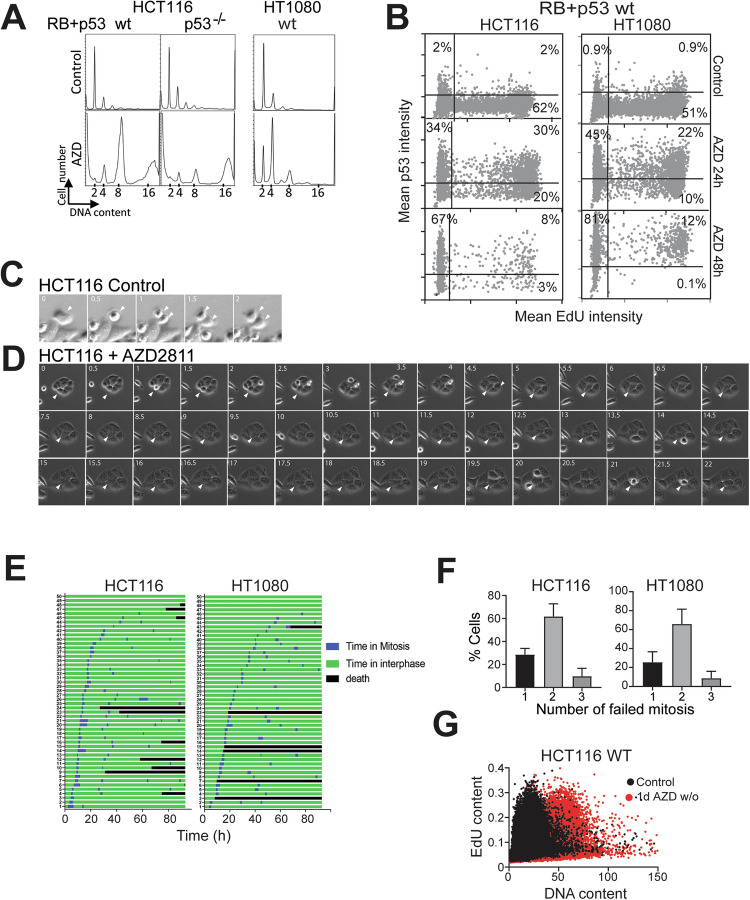
P53-induced cell cycle arrest occurs after 2 failed cytokinesis. **A** RB+p53 wild type (wt) HCT116 and HT1080, and p53 deleted HCT116 cell lines were treated without (Control) or with 0.2 μM AZD2811 for 48 h then harvested for flow cytometry of DNA content. The data is representative of three independent experiments. **B** HCT116 and HT1080 cells either untreated (Control) or treated with AZD2811 for 24 or 48 h were labelled with EdU for 2 h then fixed and stained for EdU incorporation and p53, then >1000 cells/well in triplicate were analysed by high content image analysis. The data represents the pooled wells. The level of EdU and p53 staining in each cell is shown and percentage of cells in each quadrant shown. **C**, **D** HCT116 wild type cells were either untreated (Control) or treated with 0.2 μM AZD2811 then followed by time lapse microscopy, imaging every 30 min. A control mitosis and cytokinesis is shown (**D** white arrowheads) and two failed division in AZD2811-treated cells (**D** white arrow heads). **E** HCT116 and HT1080 cells were treated with 0.2 μM AZD2811 and followed by time lapse microscopy. Timelines for 50 cells in each cell line are shown, with the time in mitosis shown. All cells failed cytokinesis. **F** Analysis of time lapse imaging of AZD2811 treated HCT116 and HT1080 cells to quantify the number of mitoses each cell underwent over 80 h of treatment. Data are mean and SD for 100 cells were followed for each cell line from three independent experiments. **G** Cells from the control and cultures treated with AZD2811 for 1 day then drug washed out, were allowed to proliferate for a week. The resultant cultures were pulsed with EdU for 2 h to identify the proliferating population, and then fixed, stained and >1000 cells/well in triplicate were analysed by high content image analysis. The data represents the pooled wells. The EdU incorporation and DNA content for the control and AZD2811-treated cultures was overlaid.

The consequence of the cell cycle arrest in the AZD2811 treated RB and p53 proficient cells is senescence [19], although the contribution of RB and p53 to this outcome is controversial [26]. In normal proliferating cells, failure of cytokinesis has been reported to trigger a cell cycle checkpoint arrest termed the tetraploidy checkpoint that is controlled by p53 [40]. However, AZD2811-treatment of RB+p53 wild type HCT116 colon cancer cells, HT1080 fibrosarcoma cells and normal neonatal foreskin fibroblasts showed accumulation of cells with >4n DNA content which should not be possible if the p53-dependent tetraploidy checkpoint functioned as modelled (Figs. 2A and S3A). Using high content imaging of p53 and EdU, it was found that >60% of RB+p53 wild type HCT116 and HT1080 cells had elevated p53 levels after 24 h of AZD2811 treatment, although half of these were EdU labelled (Fig. 2B). The proportion of cells expressing p53, but not the level of p53, increased modestly by 48 h when EdU positive cells were strongly reduced. Primary neonatal foreskin fibroblasts treated with AZD2811 showed a similar delayed loss of EdU incorporation despite the elevated p53 and >4n DNA at 24 h (Fig. S3A, B). The delayed cell cycle arrest was confirmed by time lapse microscopy of HCT116 and HT1080 cultures. AZD2811-treated mitotic cells fail to undergo cytokinesis, instead re-entering interphase without division (Fig. 2C, D). Timelines for individual cells revealed that AZD2811-treatment resulted in all cells failing cytokinesis and continued to undergo on average two failed cytokinesis in the first 48 h of treatment (Fig. 2E, F). The same accumulation of cells with >4n DNA content was observed in human cervical keratinocytes (HCK) stably expressing TERT [37] with 48 h AZD2811 treatment, although expression of HPV E6/E7 permitted further increase in ploidy (Fig. S3C). The contribution of p53 to the arrest after two failed cytokinesis is evident in p53 knockout HCT116 cells that accumulated with significantly higher ploidy after AZD2811 treatment (Fig. 2A). These data predict that cells that fail only one cytokinesis to become tetraploid should be able to continue to proliferate in the tetraploid state. HCT116 cells were treated with AZD2811 for only 24 h, then the drug removed, and the cells allowed to recover for a week in culture. These cells had increased ploidy but retained proliferative activity assessed by EdU labelling similar to controls but had twice the DNA content (Fig. 2G). These data demonstrate that p53-dependent cell cycle arrest does not occur immediately after a single failed cytokinesis but requires at least two failed cytokinesis to inhibit further endomitotic cycles.

### Requirement for RB and p53 in AZD2811-induced senescence

**Fig. 3:**
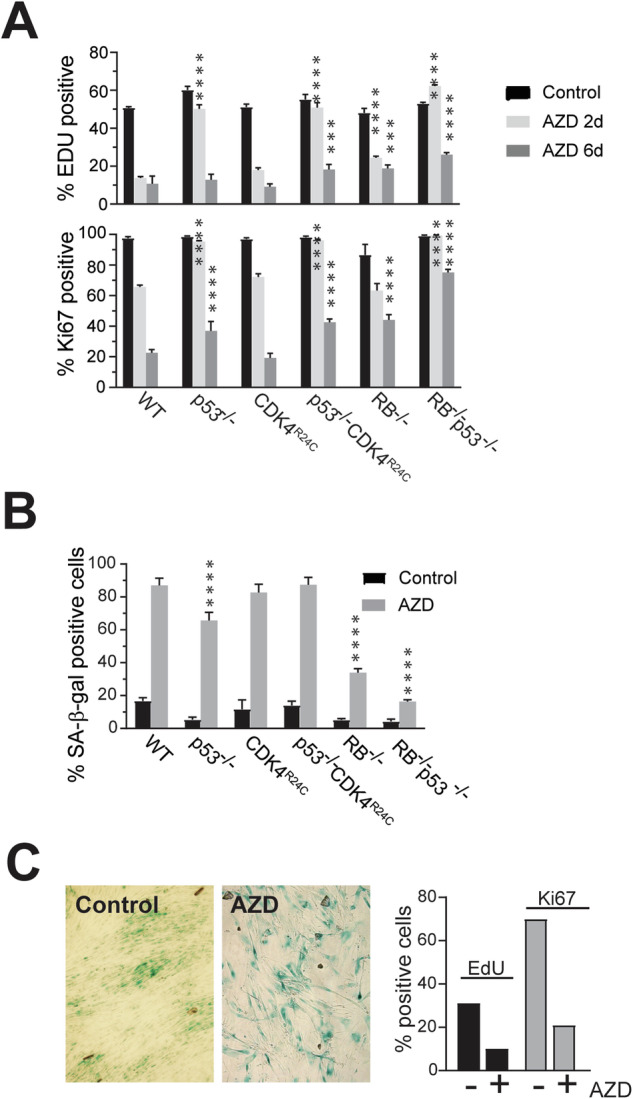
Only RB is indispensable for AURKBi-induced senescence. **A** HCT116 cells with indicated genotypes were treated with 0.2 μM AZD2811 for two or six days. Cells were labelled with EdU for 2 h then fixed. Cells were then stained for EdU and Ki67 and >1000 cells per well were quantitated by high content imaging. The data represent the mean and SD of four replicates from at least two independent experiments. **B** Cells treated as in (**A**) were fixed and stained for senescence associated β-galactosidase activity (SA-β-Gal). Quantitation of the SA-β-Gal positive cells in the indicated HCT116 genotypes. The data are the mean and SD from quantitating >four fields per cell line, each with >100 cells/field. Comparisons were to the equivalent treatment point in the WT control using two-way ANOVA with Tukey’s multiple comparison test. *** *p* < 0.001, ****p < 0.0001. **C** NFF cells were treated as in (**A**) then either stained for SA-β-Gal or labelled with EdU and Ki67 then >2000 cells analysed by high content imaging.

RB and p53 function is critical for cell cycle arrest after treatment with AZD2811 (Fig. 1C). However, the individual contribution of RB and p53 or their signalling pathways was unclear. To investigate this, a panel of HCT116 cells deleted for RB or p53, or RB pathway defect (expression of CDK4^R24C^ mutant that bypasses p16INK4A-induced senescence [41]) was used. AZD2811 treatment for 2 days resulted in reduced EdU and Ki67 staining which was further reduced by 6 days in the wild type cell line, corresponding to the majority cells being positive for the SA-β–Gal (Fig. 3A, B). Loss of p53 resulted in little decrease in EdU or Ki67 staining at 2 days (Fig. 3A), but by 6 days, cells had reduced EdU and Ki67 levels and increased SA-β–Gal similar to wild type cells (Fig. 3A, B). Expression of CDK4^R24C^ had no significant effect, and loss of both pathways had little more effect than p53 deletion alone (Fig. 3A, B). Deletion of RB had a modest although significant effect on EdU incorporation at 2 days, and significantly higher EdU and Ki67 levels at 6 days and reduction in the SA-β–Gal positive cells. Deletion of both RB and p53 resulted in the least changes in any of the parameters even at 6 days (Fig. 3A, B). NFF cells treated with AZD2811 also had reduced EdU and Ki67 staining and were SA-β–Gal positive (Fig. 3C).

### Loss of p53 delays but does not prevent repression of critical cell cycle regulators

**Fig. 4:**
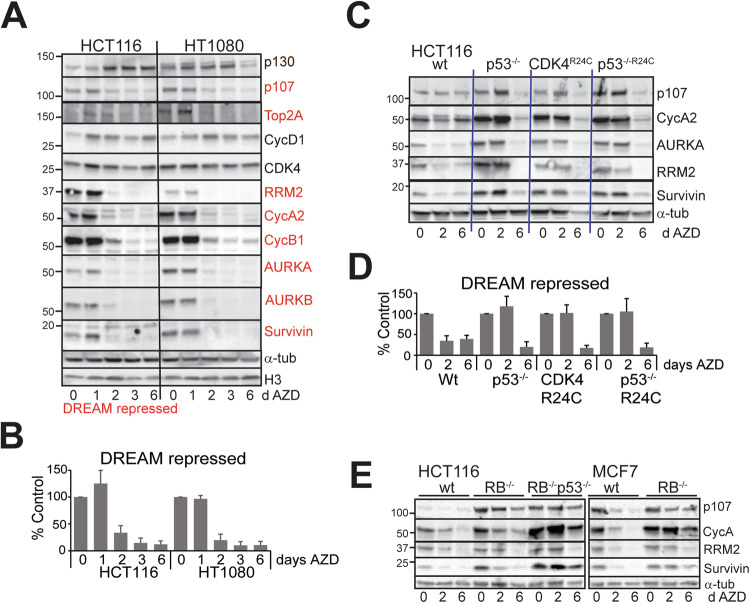
Loss of p53 only delays downregulation of critical cell cycle proteins. **A** RB and p53 wild type HCT116 and HT1080 cells were treated for the indicated number of days with 0.2 μM AZD2811, then harvested and lysates immunoblotted for the indicated proteins. DREAM repressed cell cycle regulators (defined in [23]) are highlighted in red. This is representative of two independent experiments. **B** The levels of the DREAM repressed cell cycle regulators in (**A**) were quantified, expressed as percentage of control and combined. **C** The indicated HCT116 genotypes were treated for the indicated times with 0.2 μM AZD2811, harvested and lysates immunoblotted for the indicated proteins. The data are representative of replicate immunoblots. **D** The combined changes in cell cycle regulators from (**C**). **E** HCT116 wild type, RB deleted and RB+p53 deleted, MCF7 wild type and RB deleted lines were treated for the indicated times with 0.2 μM AZD2811 and immunoblotted for the indicated proteins. α-Tubulin was used as a loading control.

Major drivers of senescence are inhibition of E2F-driven gene expression and activation of the DREAM repressor complex that control the expression of critical cell cycle regulators of progression into and through S and G2/M phase [23, 25]. There is a high level of overlap in the genes regulated by these two mechanisms [23, 25]. DREAM repressor complex is regulated by p53 through p21WAF1 expression inhibiting CDK2 activity [23, 42]. We examined the levels of a panel of critical cell cycle regulators that are known DREAM targets (indicated in red, Fig. 4A) [23]. AZD2811 treatment resulted in reduced levels of all these proteins in RB+p53 wild type HCT116 and HT1080 cell lines (Fig. 4A) with similar kinetics of reduction at 2 days treatment (Fig. 4B). The RB-related p130 which has a preferred role in p53-regulated G1 phase DREAM repressor activity [43] increased (Fig. 4A). Deletion of p53 or overexpression of CDK4^R24C^ blocked the reduction of the cell cycle regulators at 2 days, but the levels were reduced to comparable with AZD2811-treated wild type cells by day 6 (Fig. 4C, D). The delayed reduction in cell cycle regulators targets was also observed in RB wild type p53 mutant/deleted NSCLC and melanoma cell lines, corresponding to cells exiting the cell cycle by 6 days indicated by the reduced Ki67 and EdU levels (Fig. S4A, B). Deletion of RB in HCT116 cells blocked the reduction of cell cycle regulators at 2 days treatment and the level of these targets was above the wild type cells at day 6. Loss of RB+p53 in HCT116 and MCF7 cells (MCF7 are p53 wild type) abrogated the reductions (Fig. 4E). This lack of effect on cell cycle regulators and Ki67 staining was also observed in two RB+p53 defective SCLC cell lines although the cells had clearly failed cytokinesis repeatedly indicated by large nuclear size (Fig. S4C, D). Together these data indicate that loss of p53 alone only delayed the cell cycle exit and senescence, but loss of both RB and p53 effective bypassed the senescence induced by AZD2811.

### AURKBi-induced dephosphorylation of RB is independent of the CDK activity

**Fig. 5:**
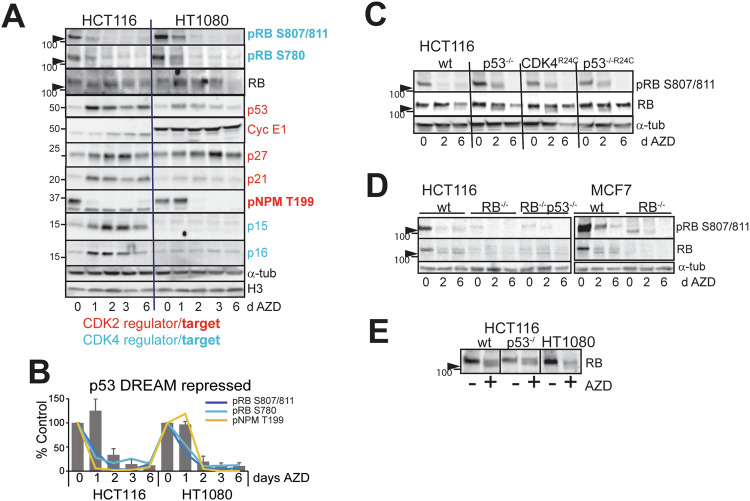
AURKBi downregulates CDK4 and CDK2 activity. **A** HCT116 and HT1080 cells were treated for the indicated times with 0.2 μM AZD2811, then harvested and lysates immunoblotted for the indicated markers of CDK2 and CDK4 activity. The high mobility hypophosphorylated form of RB is indicated by the arrowhead in all panels. This is representative of replicate experiments. **B** The levels of phospho-RB and phospho-NPM were superimposed on the changes in the repressed cell cycle regulators (Fig. 4B). **C** The indicated HCT116 genotypes were treated for two or six days with 0.2 μM AZD2811 as in Fig. 4C and immunoblotted for RB and phospho-RB. **D** HCT116 wild type, RB deleted and RB and p53 deleted, MCF7 wild type and RB deleted lines were treated for the indicated times with AZD2811 and immunoblotted for the indicated proteins. The high mobility hypophosphorylated form of RB is indicated by the arrowhead. **E** HCT116 wild type and p53^−/−^, and HT1080 cells were treated without (Con) or with 0.2 μM AZD2811 for two days then harvested for immunoblotting for RB.

AZD2811 treatment resulted in rapid dephosphorylation of CDK4-dependent sites RB Ser780 and Ser807/811, corresponding the appearance of faster migrating hypophosphorylated form of RB (Fig. 5A). This suggested decreased CDK4/Cyclin D activity with treatment. The levels of CDK4/6 inhibitory subunits p15^INK4B^ and p16^INK4A^ increased in HCT116 but not HT1080 cells where the *CDKN2A/B* locus is deleted [44] (Fig. 5A). The levels of CDK4 and Cyclin D1 were relatively unchanged (Fig. 4A), suggesting that AURKBi triggered other mechanisms to regulate RB phosphorylation. The accumulation of p53 and p21, modest accumulation of Cyclin E and p27 [45, 46] and loss of CDK2/Cyclin-dependent NPM Thr199 phosphorylation [47], all indicate inhibition of CDK2/cyclin (Fig. 5A). The reduction in CDK4 and CDK2-dependent phosphorylation immediately preceded cell cycle regulator reduction (Fig. 5B). Deletion/mutation of p53 or over-expression of CDK4^R24C^ modestly delayed dephosphorylation of the CDK4 sites on RB, but the hypophosphorylated form was present in all genotypes with treatment (Figs. 5C–E and S4A, B). The appearance of hypophosphorylated RB was triggered by other AURKB inhibitors to a similar extent as AZD2811 in the RB+p53 wild type HCT116 and U2OS cell lines (Fig. S5), indicating that this was a consequence of AURKB inhibition and not an off-target effect of AZD2811. We have also demonstrated that the faster migrating hypophosphorylated RB band [48] is not a consequence of proteolytic cleavage of the C-terminal region of RB by either caspases or calpain [49, 50] (Fig. S6).

**Fig. 6:**
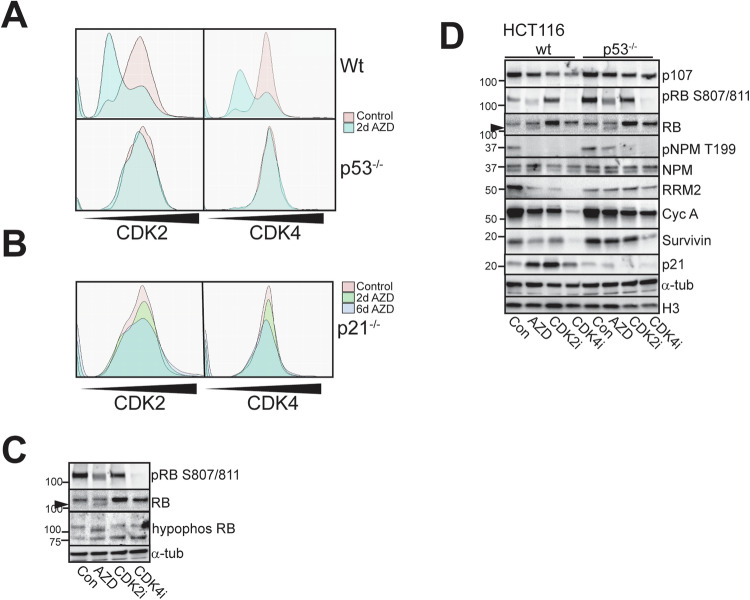
RB hypophosphorylation is independent of CDK4 and CDK2 activity. **A** HCT116 wild type and p53^−/−^ cells expressing both CDK2 and CDK4 biosensors were treated as indicated for two days then biosensor localisation was assessed by high content imaging and the activity calculated by determining the ratio of cytoplasmic to nuclear biosensor fluorescence. **B** HCT116 p21^−/−^ cells expressing both biosensors were treated and analysed as in (**A**). For each experiment >2000 cells were analysed. The data are the average of triplicate experiments. **C** HCT116 cells, either untreated or treated with AZD2811 or 10 μM CDK2 or CDK4 selective inhibitors for two days were harvested and immunoblotted with indicated antibodies. **D** The indicated HCT116 genotypes were treated for two days with either 0.2 μM AZD2811 or 10 μM CDK2 or CDK4 inhibitors and cell lysates immunoblotted for the indicated markers.

These data indicate that RB hypophosphorylation was induced by AURKB inhibition, suggesting AURKB inhibitors might downregulate CDK2 and/or CDK4 activity responsible for RB hyperphosphorylation. To assess CDK2 and CDK4 activity directly, HCT116 wild type, p53^−/−^ and p21^−/−^ cells were transduced with biosensors of CDK2 and CDK4 activity [31]. Two days AZD2811 treatment reduced CDK2 and CDK4 activity in the wild type cells but not p53^−/−^ cells (Fig. 6A). The activity of CDK2 and CDK4 remained low at 6 days in the wild type cells but was only modestly reduced in the p53^−/−^ cells (Fig. S7). The surprising lack of CDK4 inhibition in p53^−/−^ cells with AZD2811 was overcome with a CDK4 inhibitor (Fig. S7). The lack of AZD2811-induced CDK2 and CDK4 inhibition was also observed in p21^−/−^ cells (Fig. 6B), suggesting AZD2811-induced p53 p21 expression contributed significantly to CDK4 inhibition.

The faster migrating RB band was confirmed as the hypophosphorylated form using an antibody that selectively detects hypophosphorylated RB. This strongly detected the higher mobility band in the AZD2811-treated cells, but this band was not present in cells treated for 2 days with either CDK2 or CDK4 inhibitors, even though the CDK4 inhibitor effectively inhibited RB Ser807/811 phosphorylation. This result suggested the hypophosphorylated RB was not simply a result of inhibition of CDK2 and CDK4 (Fig. 6C).

The reduction of CDK2 and CDK4 activity observed in AZD2811-treated RB+p53 functional cells indicated that CDK4 and CDK2 inhibition were required for the efficient cell cycle exit into senescence. Inhibition of CDK4 that effectively inhibited RB Ser807/811 phosphorylation also inhibited the CDK2-dependent NPM Thr199 phosphorylation (Fig. 6D). CDK4 inhibitors have been demonstrated to promote senescence through a p53-p21dependent mechanism [51], and CDK4 inhibitor failed to trigger p21 accumulation in the p53^−/−^ cells (Fig. 6D). CDK4 inhibitor was less effective in reducing the cell cycle regulators in p53^−/−^ cells, indicating that both CDK2 and CDK4 inhibition are required for full target repression. This was further evidence that the ability of AURKBi to promote RB hypophosphorylation was independent of its ability to inhibit CDK2 and CDK4 activity.

## Discussion

This work has investigated the mechanism by which AURKBi promotes senescence. We have demonstrated that loss of RB through mutation, deletion and inactivation by viral oncogenes, but not through defects in the RB-CDK4-p16 pathway, were essential for bypass of the AURKBi-induced senescence. This supports the reports that the AURK inhibitors promoted senescence in a high proportion of melanoma cell lines [21] where the majority have RB pathway signalling defects but few have RB loss of function mutations or deletion [52]. Mutation or deletion are the most common means of inactivation of the p53 pathway in cancers. Although RB and p53 pathways have been long understood to be key drivers of senescence in response to a wide range of stimuli [22, 27], the mechanism by which they contribute to AURKBi-induced senescence was unknown.

**Fig. 7:**
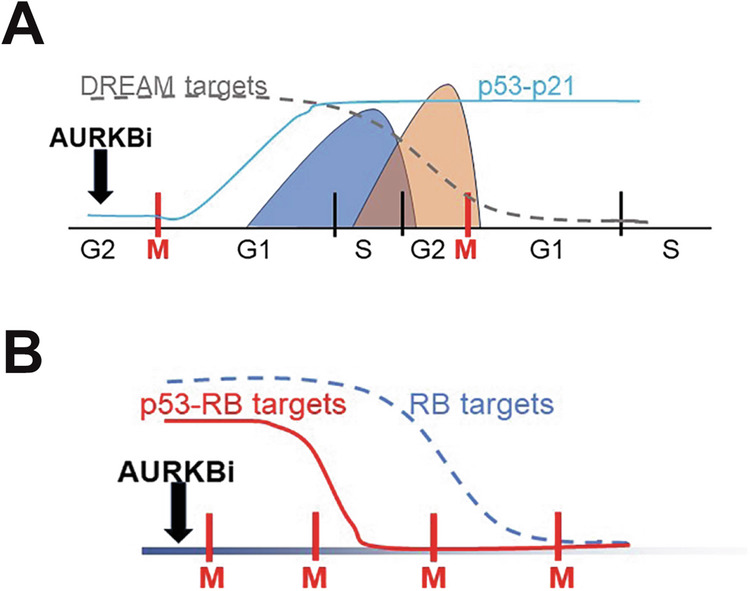
Model for how p53 imposes a cell cycle arrest after two failed cytokinesis in AURKBi-treated cells. **A** P53 is induced after the first failed cytokinesis but this is too late to block S phase progression or impose the DREAM repression of S/G2/M phase genes. However, these genes are cell cycle regulated at both mRNA and protein levels and will be downregulated after the second failed cytokinesis. **B** AURKBi imposed senescence by both increased p53 activity and hypophosphorylation of RB resulting repression of the overlapping RB and DREAM repressor gene expression. In the absence pf p53, hypophosphorylation of RB is sufficient to promote senescence albeit this is delayed.

We have demonstrated that p53 is activated in response to a single failed cytokinesis as reported previously [7, 53], but this is not effective in promoting a cell cycle arrest until cells had undergone 2 failed cytokinesis. It appears that the inhibition of CDK2 activity through increased p21 was not sufficient before the first S phase after failed cytokinesis to block progression into and through the remainder of the cell cycle, but requires the combination of inhibition of CDK2 activity and DREAM repression of cell cycle regulators of progression into S phase and mitosis [23]. The destruction of these regulatory proteins did not occur until 48 h, that is after 2 failed cytokinesis, indicating that even when transcription has been repressed cells must destroy their stocks of cell cycle regulator protein [54]. This occurs after transit through another cell cycle and failed mitosis as experimentally observed (Fig. 7A). Surprisingly, loss of p53 only delayed the onset of cell cycle arrest and senescence, likely to be through the lack of p53-p21-induced DREAM complex repression [23]. However, even in the absence of p53, RB was driven into the hypophosphorylated repressor state by AURKBi, thereby inhibiting E2F activity which would downregulate the many of the same cell cycle regulators [23] albeit more slowly than DREAM repressor (Fig. 7B).

RB deletion/mutation without p53 mutation/loss of function is very rare in cancers (<2% all cancers TCGA). The co-occurrence of RB and p53 deletion/loss of function mutation is present in >5% of all tumours, and up to 20% of tumour cell lines (TCGA). The ability of AURKB inhibitors to control RB phosphorylation state and thereby activity has not been previously appreciated. AURKB has been shown to phosphorylate RB S780 in vitro and dephosphorylation of this site was reduced with an AURKB inhibition [28], however S780 is also a CDK4/Cyclin D phosphorylation site and RB is dephosphorylated after exit from mitosis including S780 [55, 56], suggesting that the loss of this phosphorylation is more likely a consequence of RB dephosphorylation rather than inhibition of AURKB-dependent phosphorylation of this site. RB binds phosphatase PP1α in G1 phase [57] and PP1 is primarily responsible for the dephosphorylation of RB [52, 55], although PP2A can also dephosphorylate RB [58, 59]. AURKB might control RB dephosphorylation by regulating the binding of PP1 to RB. AURKB controls PP1 binding to a number of proteins involved in exit from mitosis and cytokinesis by phosphorylating a site proximal to a PP1-binding consensus motif [60]. RB contains one validated PP1C binding site [61], suggesting that AURKB directly regulates PP1 binding to RB. A short linear binding motif has also been identified for B55α subunit of PP2A in RB family members Rb and p107, and B55α binding was regulated by a phosphorylation site proximal to the binding motif [62]. However, we were unable to find either PP1C or B55α associated with RB in co-immunoprecipitation experiments under a range of conditions and thus unable to validate these potential mechanisms.

In summary, the combination of p53 activation in response to failed cytokinesis, and AURKBi-induced hypophosphorylation of RB promote cell cycle exit and senescence in response to AURKBi treatment. The primary role of p53 is to determine the speed of cell cycle exit, p53 triggering cell cycle arrest after two failed cytokinesis, and in the absence of p53 cell cycle exit and senescence is delayed by several days (Fig. 7B). Loss of RB has a more profound effect and is indispensable for senescence. However, loss of RB is rare in the absence of p53 mutation/deletion, thus co-loss of RB and p53 is a common genotype in cancers that can bypass the AURKBi-induced senescence arrest.

## Supplementary information

Supplementary Information

Raw immunoblots

## Data Availability

All data generated or analysed during this study are included in this published article [and its supplementary information files].
