# Supplementary material for: Aurora B inhibitors promote RB hypophosphorylation and senescence independent of p53-dependent CDK2/4 inhibition
Source: Cell Death Dis. 2024 Nov 9;15(11):810. doi: 10.1038/s41419-024-07204-5 (PMC11550316; doi:10.1038/s41419-024-07204-5)

Vora et al., Supplementary Material

Figure S1:

The indicated cell lines where treated with either 200 nM AZD2811 (AZD), 50 nM AURKB-selective BI831266 (BI), or 100nM pan-AURK inhibitor AMG900 (AMG) for 72 h then pulse labelled with EdU for 2h fixed and stained for DNA and EdU. The DNA content (**A**) and EdU incorporation (**B**) were quantified using high content imaging analysis. More 2000 cells were imaged for each condition. The data for EdU are mean and standard deviation from 4 replicate wells. Statistical analysis was performed using one way ANOVA, the significance is compared to control **** $p<0.0005$.

Figure S2:

A. Cells were treated for six days with 0.2 μM AZD2811 then fixed and stained for senescence associated β-galactosidase activity (SA-β-Gal). Image shows HCT116 cells, either control or AZD2811 treated. Quantitation of the SA-β-Gal positive cells in the HCT116 and HT1080 cell lines. The data are the mean and SD from quantitating >four fields per cell line, each with >100 cells/field.

B. HCT116 and HT1080 cells were either untreated or treated with AZD2811 for 3 days then the culture supernatant was assessed for the indicated cytokines using cytokine bead array. The data are from duplicated determinations.

C. HCK cells stably expressing the indicated HPV E6 and E7 genes were lysed and immunoblotted for p107 and p53. α-Tubulin was used a loading control.

Figure S3:

A. NFF cells, either untreated (Control) or treated with 0.2 μM AZD2811 for the indicated time and harvested for flow cytometry of DNA content. Ploidy is shown.

B. Cells from a similar experiment to A were labeled with EdU for 2 h prior to fixing, then stained for EdU incorporation and p53, and >2000 cells were analysed by high content imaging. The proportion of p53 and EdU stained cells is shown.

C. The HCK cell lines, either wild type or expressing HPV E6/E7 were treated without (Control) or with 0.2 μM AZD2811 for 48 h then harvested for flow cytometry of DNA content.

Figure S4:

A: Non-small cell lung cancer cells lines with the indicated genotype were treated for 0, 2 and 6 days with 0.2 μM AZD2811, then harvested and lysates immunoblotted for the indicated proteins and markers. Coverslips for cells treated for 6 days were immunostained for Ki67 as a marker of proliferation and percentage of stained cells reported. This is from >1000 cells each treatment.

B: Melanoma cell line SKMEL13 was treated as in A and cell lysates immunoblotted for the indicated markers and proteins. Cells grown on coverslips were treated in parallel and labelled with EdU for 2 h prior to fixation. Cells were immunostained for EdU incorporation and Ki67.

C: RB and p53 defective small cell lung cancer cell lines H69 and H2141 were treated as in A harvested and cell lysates immunoblotted for the indicated proteins and markers.

D: H69 cells treated for 6 days in parallel with C were fixed and immunostained for Ki67 (red) and DNA (blue).

Figure S5:

RB and p53 wild type HCT116 and U2OS cells were treated with 0.2 and 1 (*) μM AZD2811, 0.1 μM AMG900 and 0.05 μM BI811283 for 24 h, then cells harvested lysed and immunoblotted for the indicated proteins. The hypophosphorylated RB is indicated by the arrowhead.

Figure S6:

A: HCT116 cells were treated for 1 day with or without 0.2 μM AZD2811, 0.2 μM AZD2811 + 20 μM Q-VD-OPh (Pan Caspase inhibitor) or 0.2 μM AZD2811 + 20 μM Z-VDVAD-FMK (Caspase 2 inhibitor) then harvested, and lysates immunoblotted for the indicated proteins and markers.

B: U2OS cells with the indicated genotypes were treated with 400 nm AZD1152-HQPA for 1 day followed by lysis and immunoblotted for the indicated proteins.

C: HCT116 cells were treated for 1 day with or without 0.2 μM AZD2811 in combination with or without 25 μM calpeptin (Calpain inhibitor), then lysed and immunoblotted for the indicated proteins.

Figure S7:

HCT116 wild type and p53$^{-/-}$ cells expressing both CDK2 and CDK4 biosensors were treated with 0.2 μM AZD2811 for two and six days or CDK4 inhibitor for two days, then biosensor localisation was assessed by high content imaging and the activity calculated by determining the ratio of nuclear and cytoplasmic biosensor fluorescence. For each experiment >2000 cells were analysed. The data are the average of triplicate experiments.

Vora et al Supplementary Figure S1

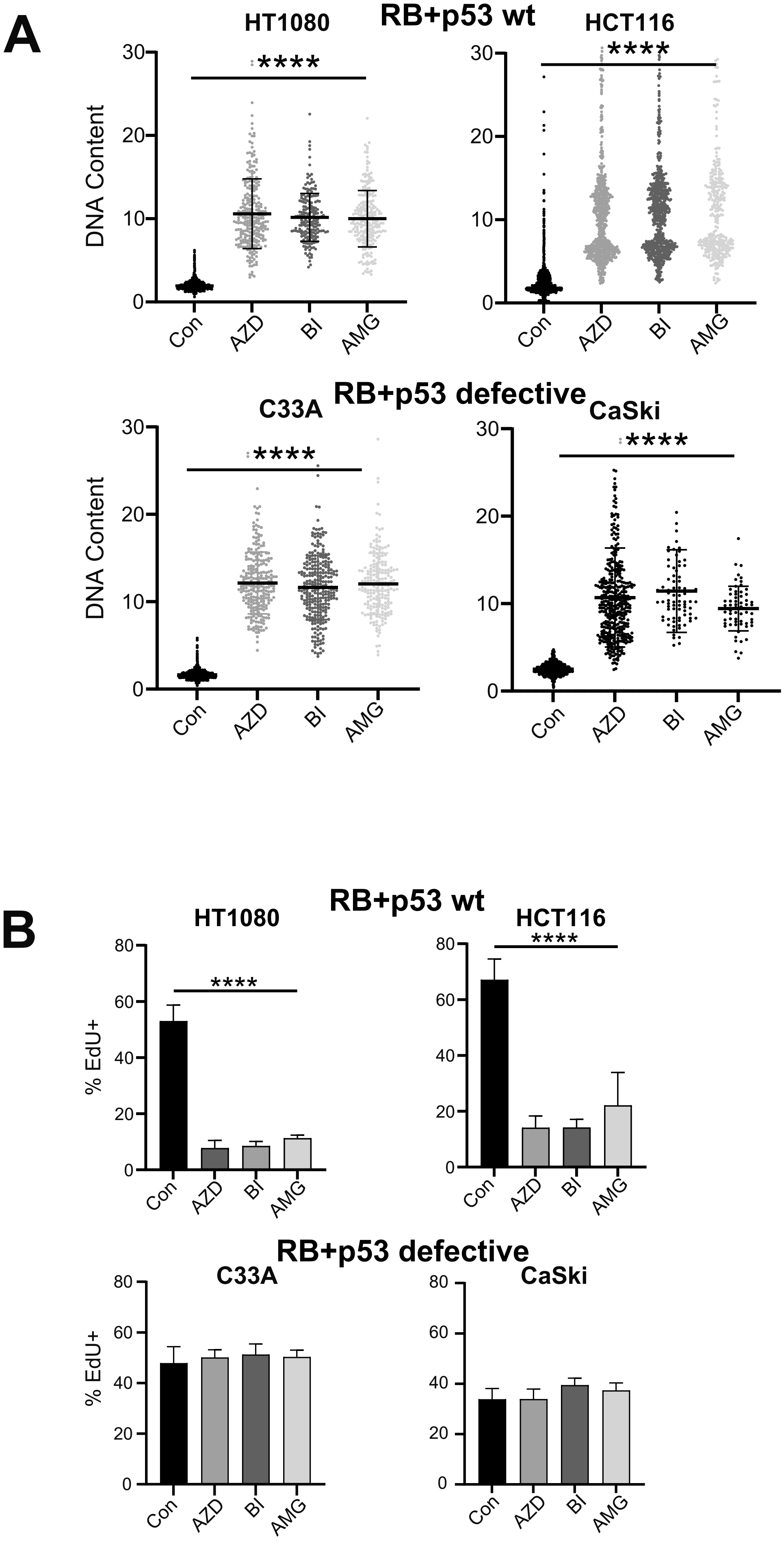

Vora et al Supplementary Figure S2

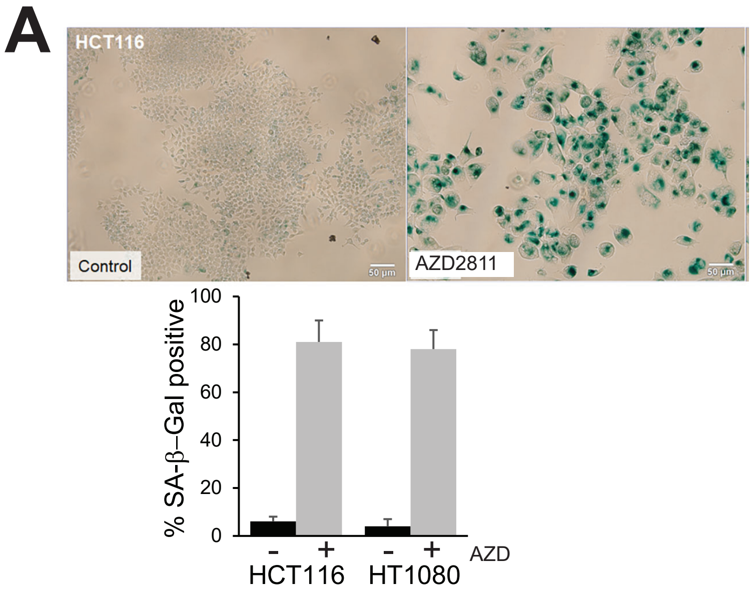

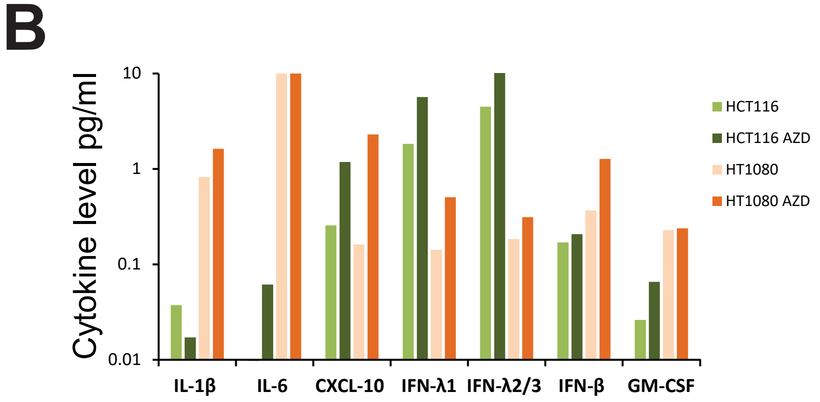

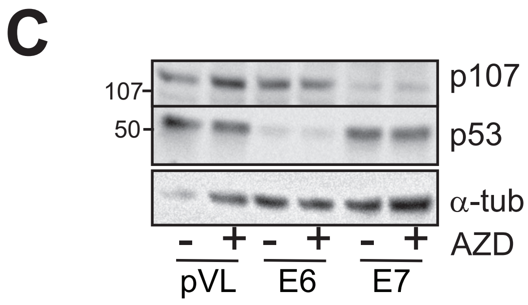

Vora et al Supplementary Figure S3

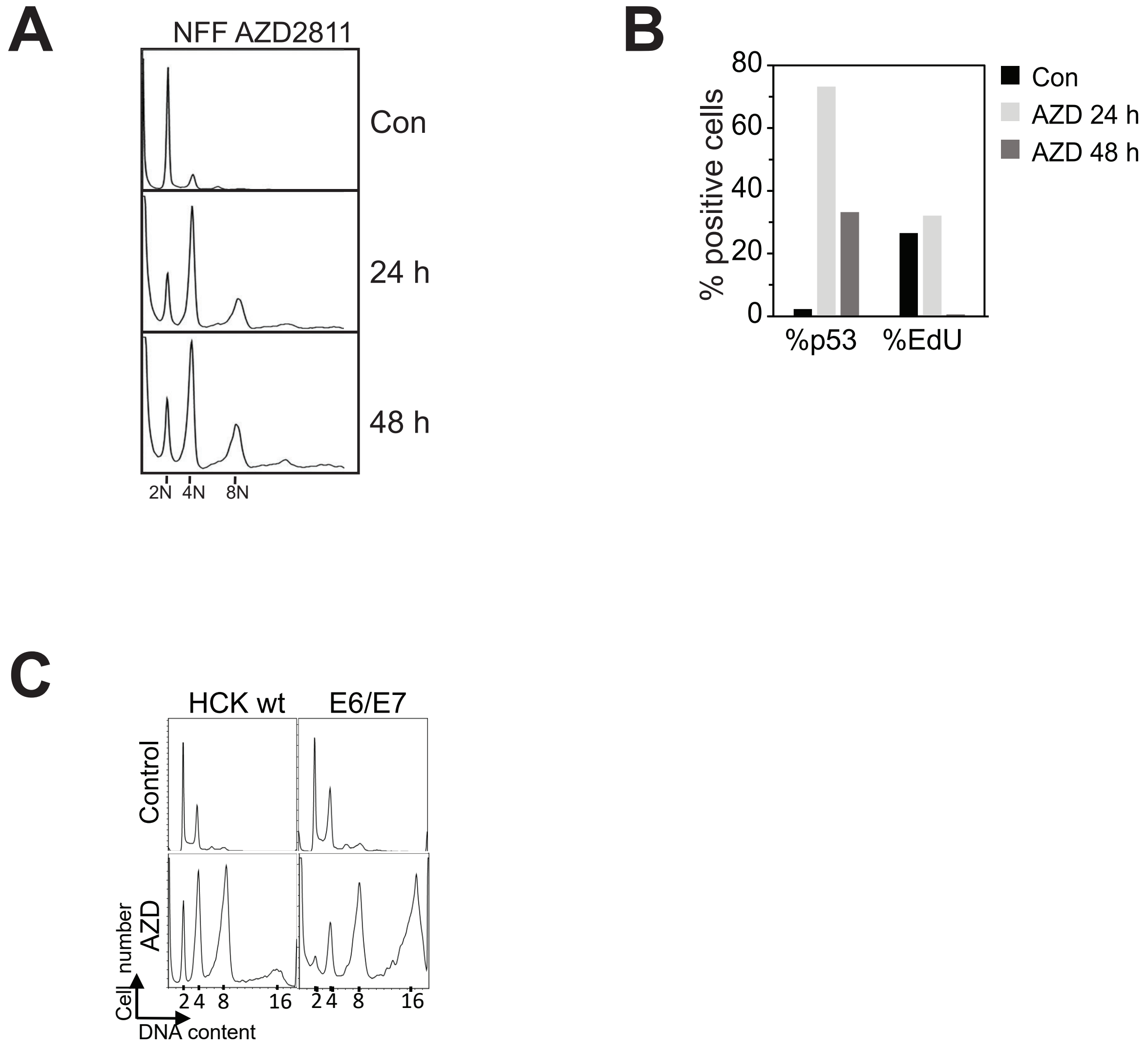

Vora et al Supplementary Figure S4

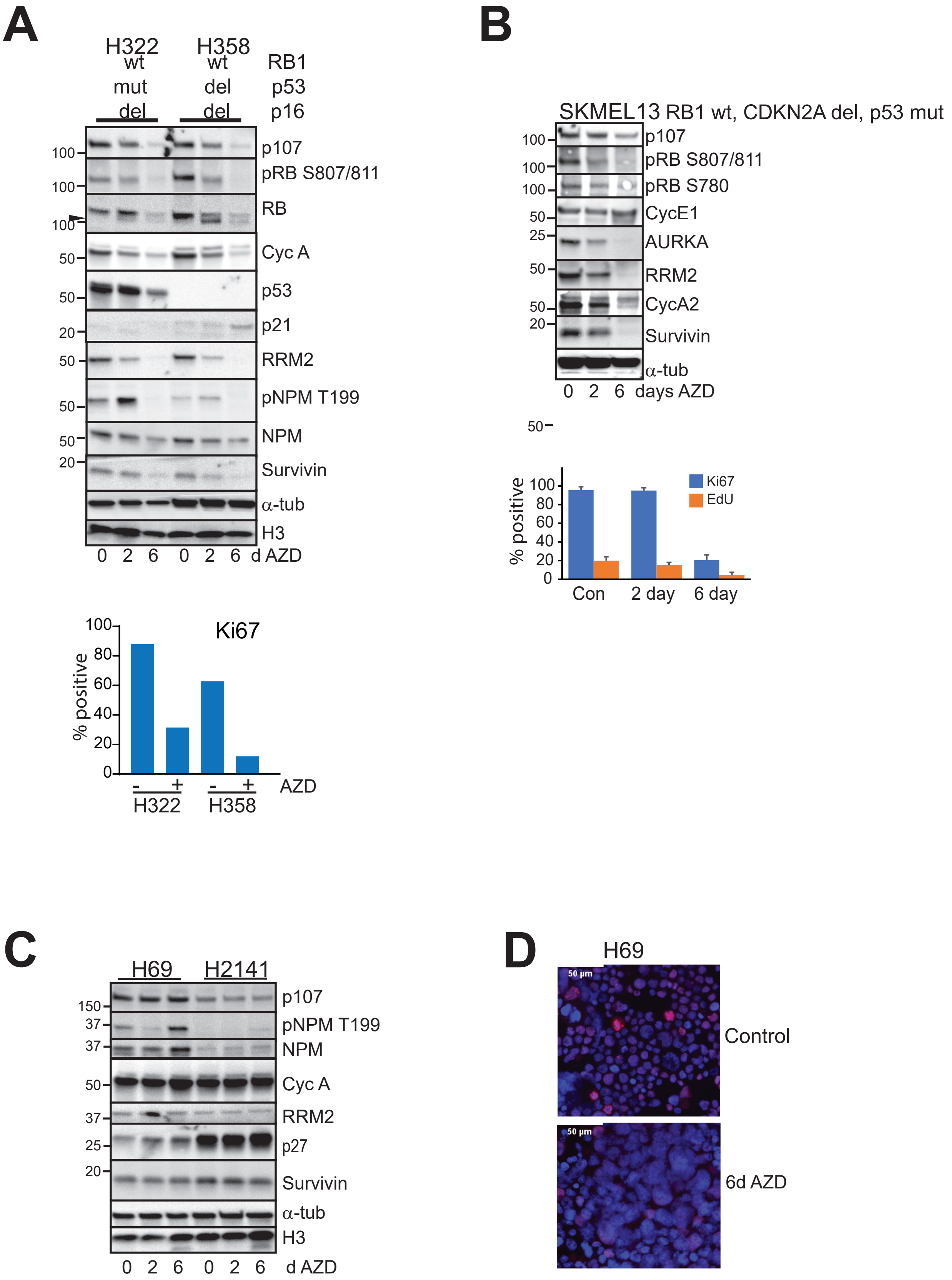

Vora et al Supplementary Figure S5

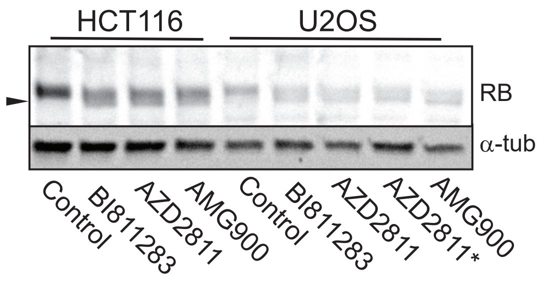

Vora et al Supplementary Figure S6

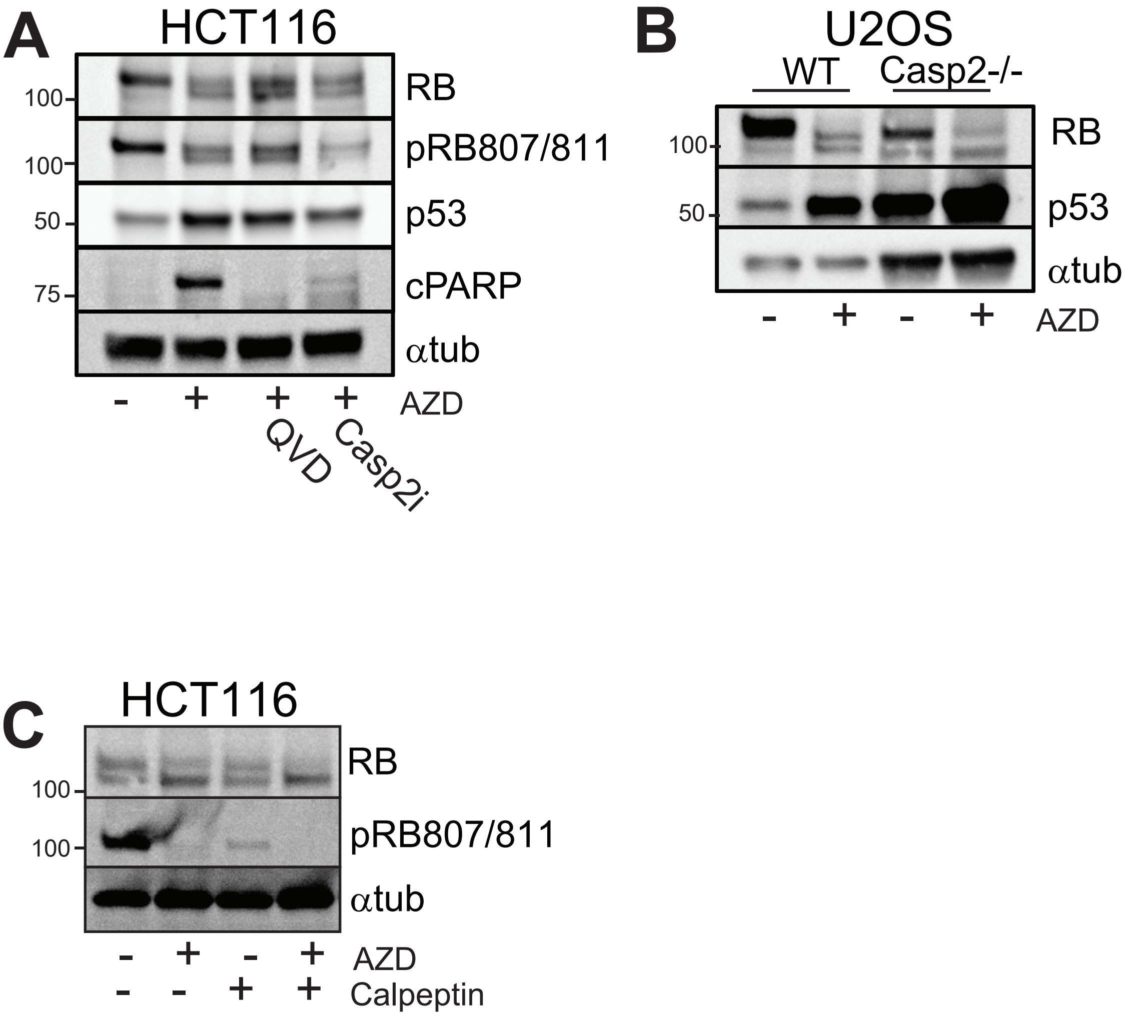

Vora et al Supplementary Figure S7

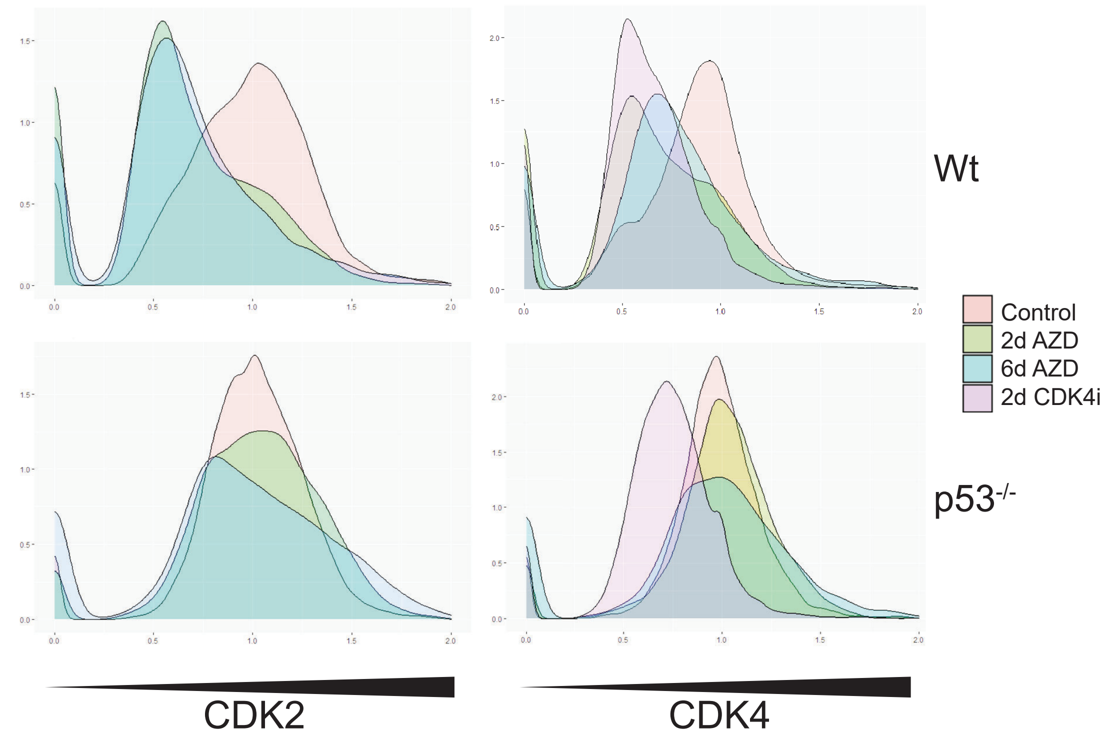

Supplement: Supplementary file 1 — Supplementary Information [file 41419_2024_7204_MOESM1_ESM.pdf]
