# Supplementary material for: Aurora B inhibitors promote RB hypophosphorylation and senescence independent of p53-dependent CDK2/4 inhibition
Source: Cell Death Dis. 2024 Nov 9;15(11):810. doi: 10.1038/s41419-024-07204-5 (PMC11550316; doi:10.1038/s41419-024-07204-5)

Figure 4A

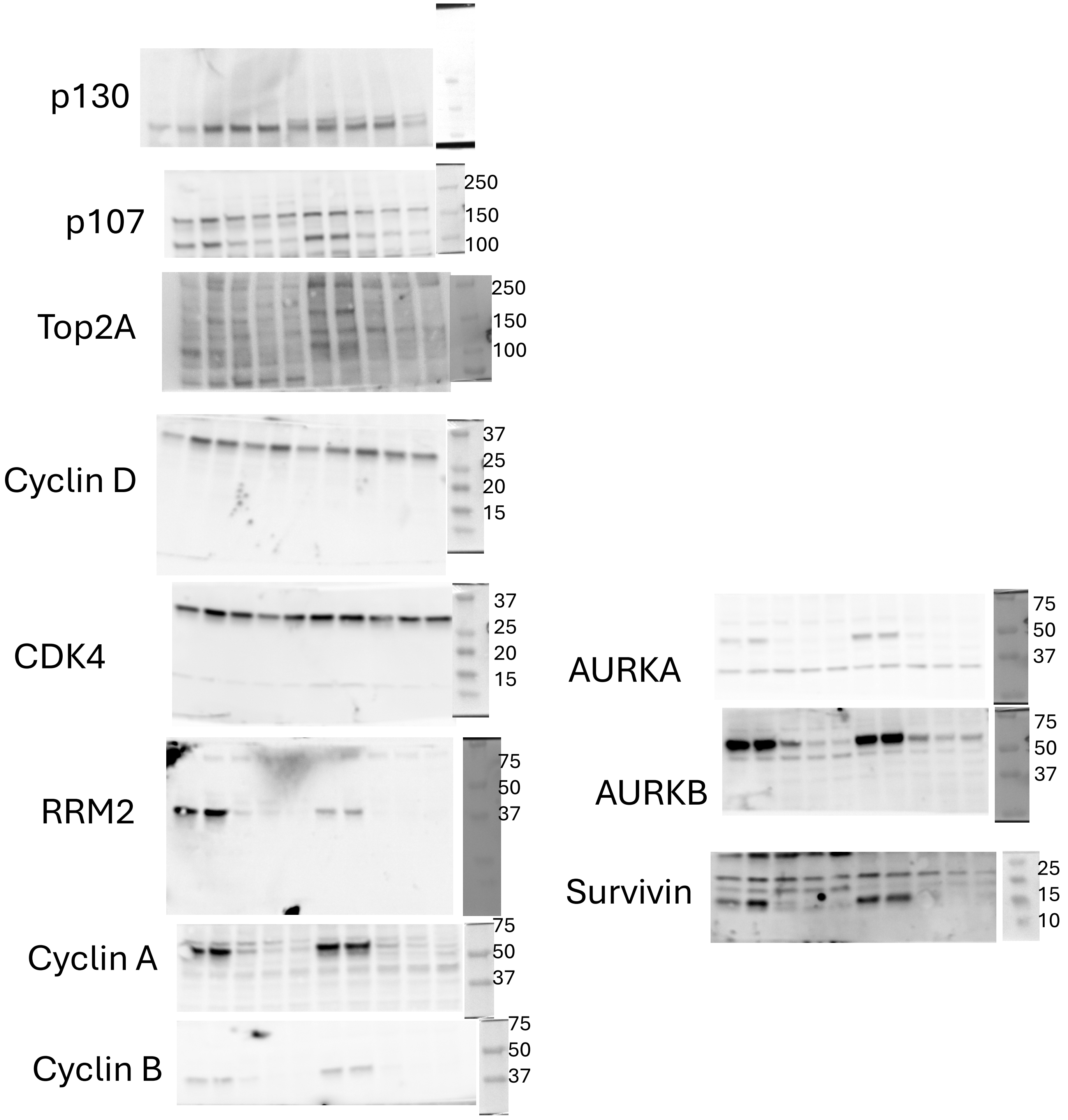
p130
p107
250
150
100
Top2A
250
150
100
Cyclin D
37
25
20
15
CDK4
37
25
20
15
RRM2
75
50
37
Cyclin A
75
50
37
Cyclin B
75
50
37
AURKA
75
50
37
AURKB
75
50
37
Survivin
25
15
10

Figure 4C

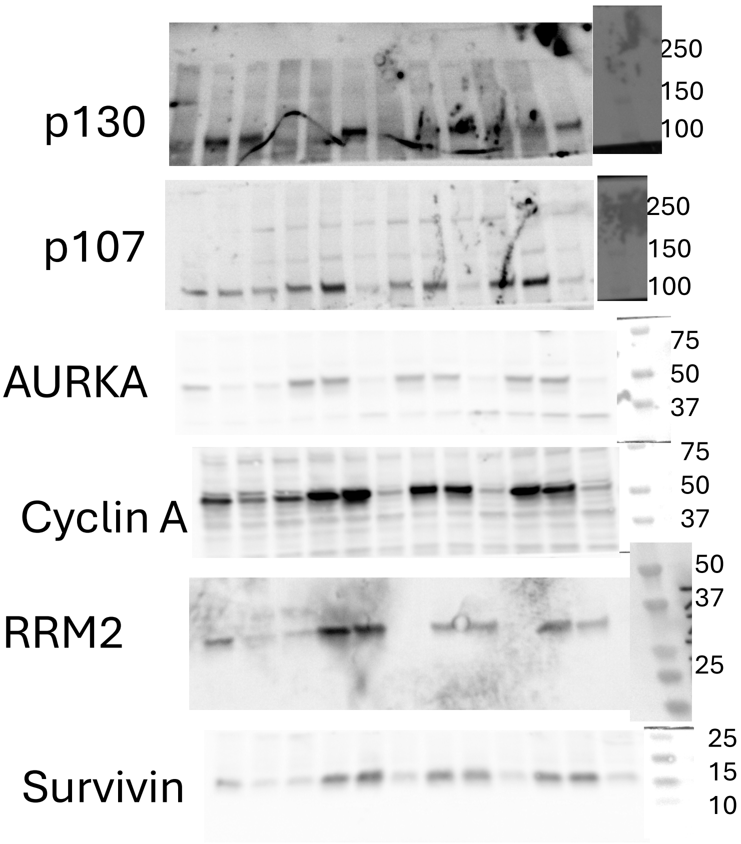

p130
250
150
100
p107
250
150
100
AURKA
75
50
37
Cyclin A
75
50
37
RRM2
50
37
25
Survivin
25
15
10

Figure 4E

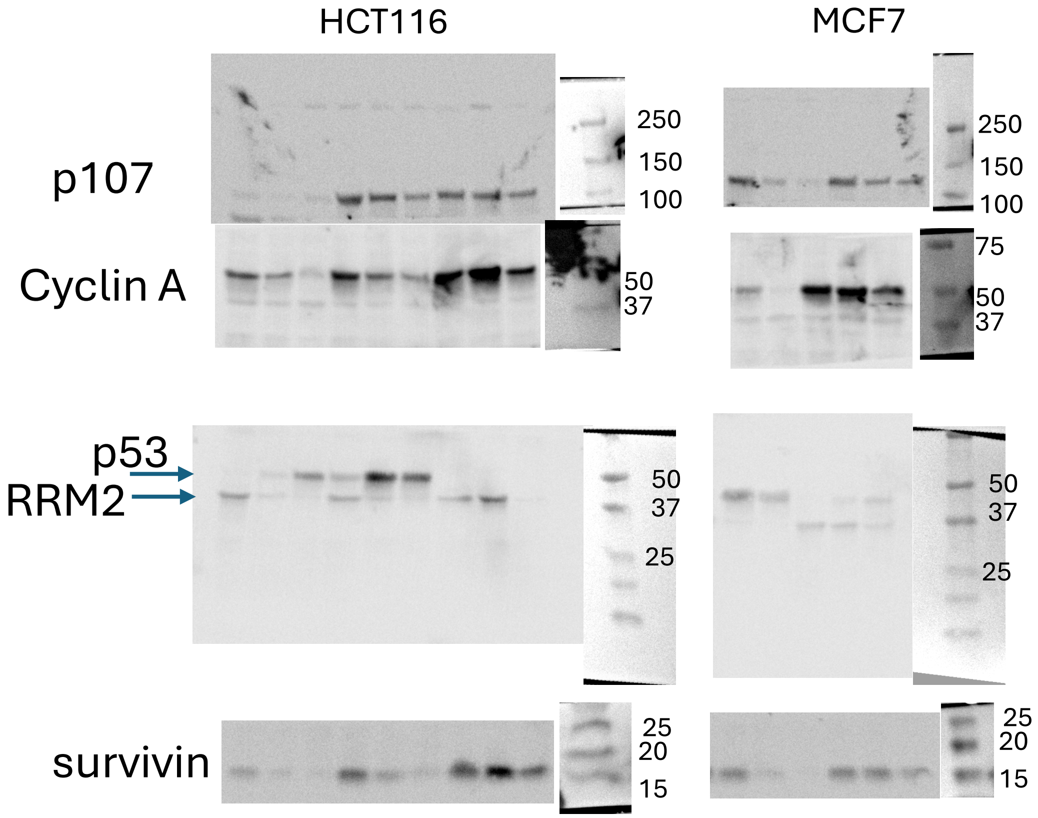
HCT116
MCF7
p107
250
150
100
250
150
100
Cyclin A
50
37
75
50
37
p53
RRM2
50
37
25
50
37
25
survivin
25
20
15
25
20
15

Figure 5A

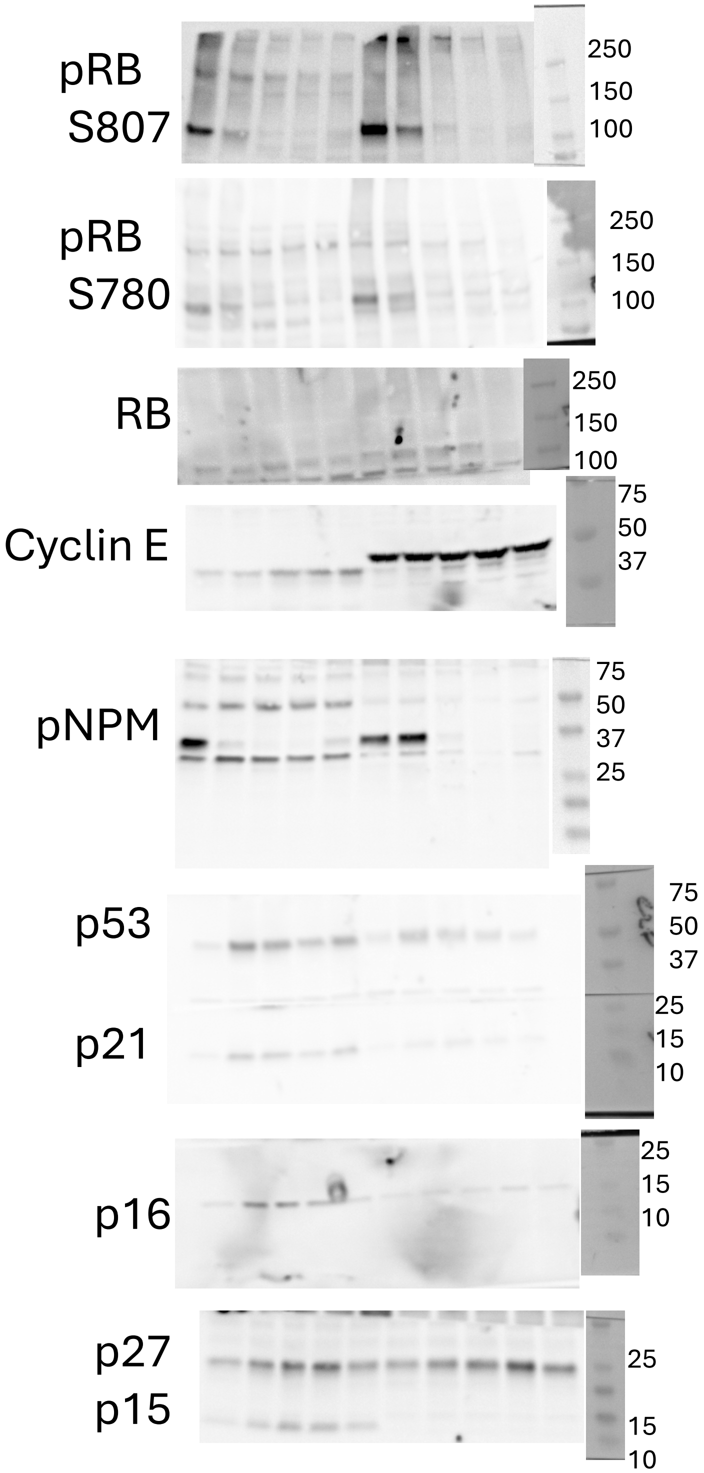
pRB S807
250
150
100
pRB S780
250
150
100
RB
250
150
100
Cyclin E
75
50
37
pNPM
75
50
37
25
p53
p21
75
50
37
25
15
10
p16
25
15
10
p27
p15
25
15
10

Figure 5C

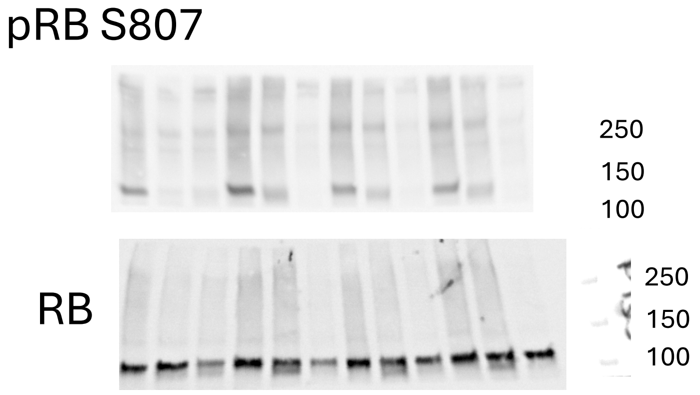

# Figure 5D

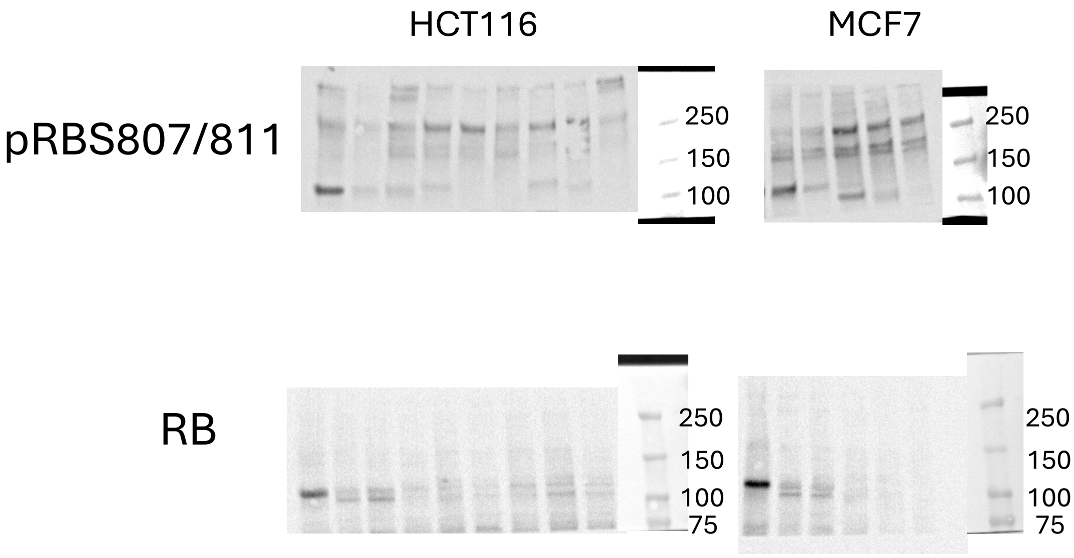


# Figure 5E

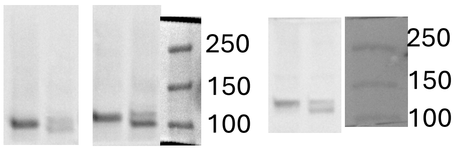

Figure 6C

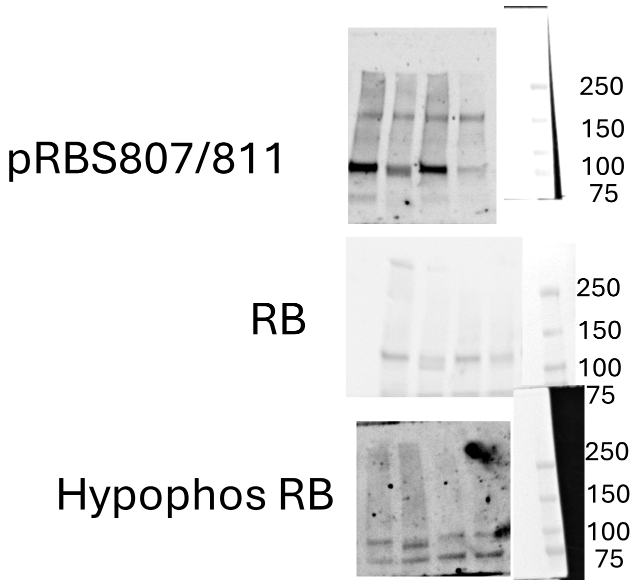

pRBS807/811
250
150
100
75
RB
250
150
100
75
Hypophos RB
250
150
100
75

Figure 6D

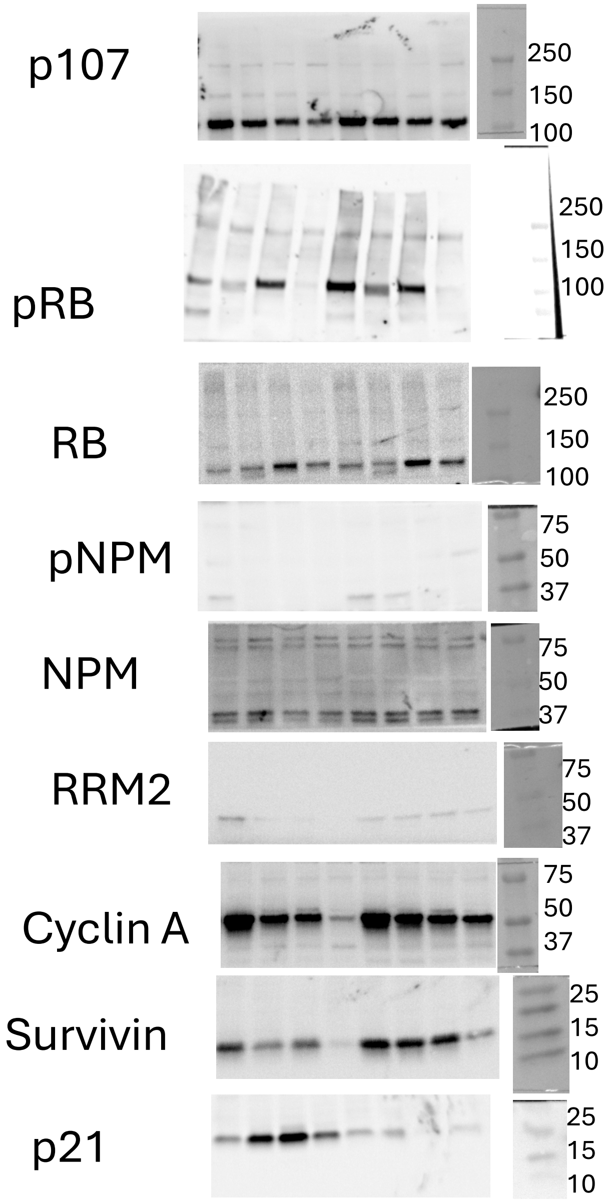
p107
250
150
100
pRB
250
150
100
RB
250
150
100
pNPM
75
50
37
NPM
75
50
37
RRM2
75
50
37
Cyclin A
75
50
37
Survivin
25
15
10
p21
25
15
10

Figure S3A

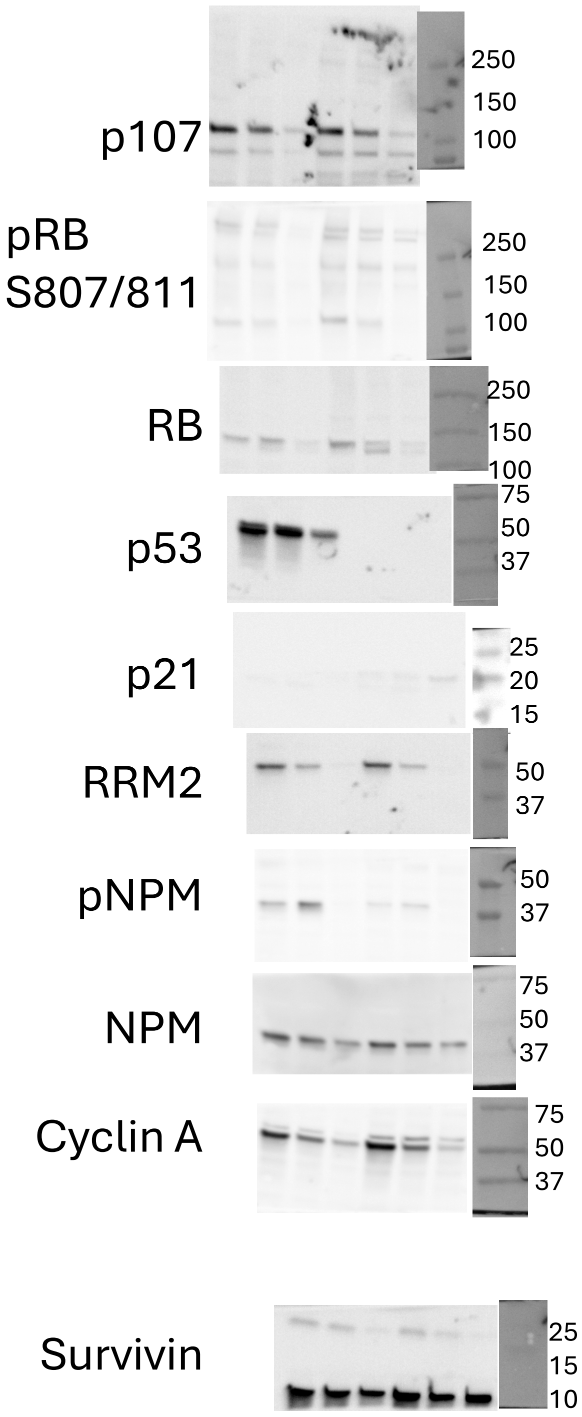

p107
250
150
100
pRB
S807/811
250
150
100
RB
250
150
100
p53
75
50
37
p21
25
20
15
RRM2
50
37
pNPM
50
37
NPM
75
50
37
Cyclin A
75
50
37
Survivin
25
15
10

Figure S3B

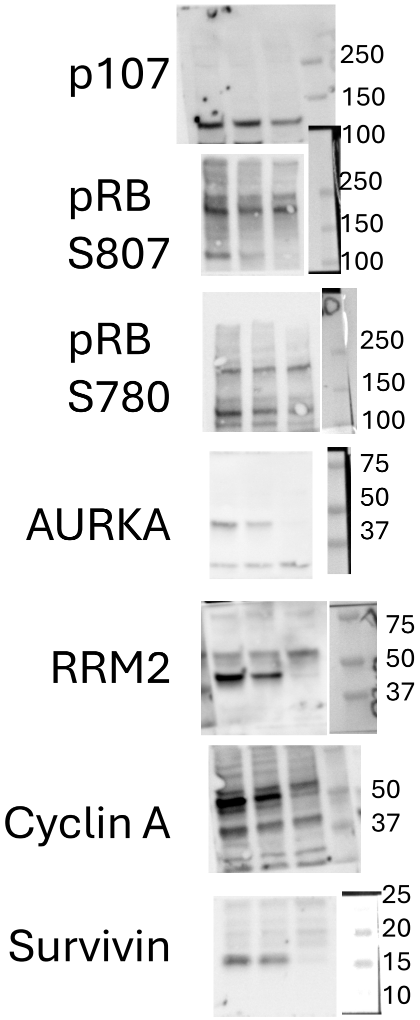

p107
250
150
100
pRB
S807
250
150
100
pRB
S780
250
150
100
AURKA
75
50
37
RRM2
75
50
37
Cyclin A
50
37
Survivin
25
20
15
10

Figure S3C

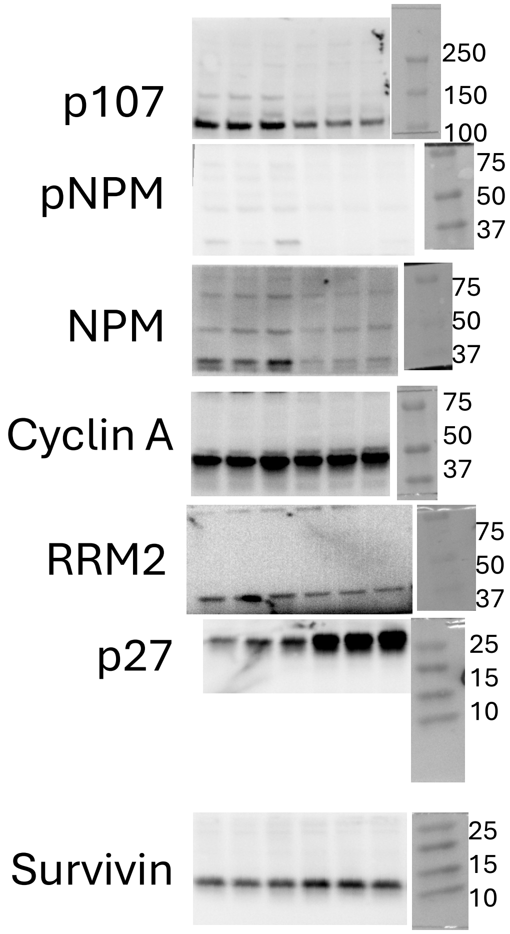
p107
250
150
100
pNPM
75
50
37
NPM
75
50
37
Cyclin A
75
50
37
RRM2
75
50
37
p27
25
15
10
Survivin
25
15
10

# Figure S4

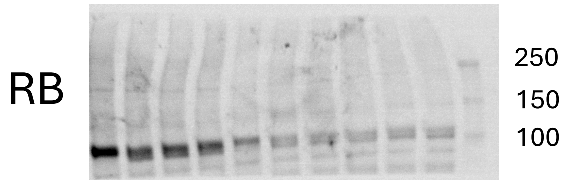


# Figure S5A

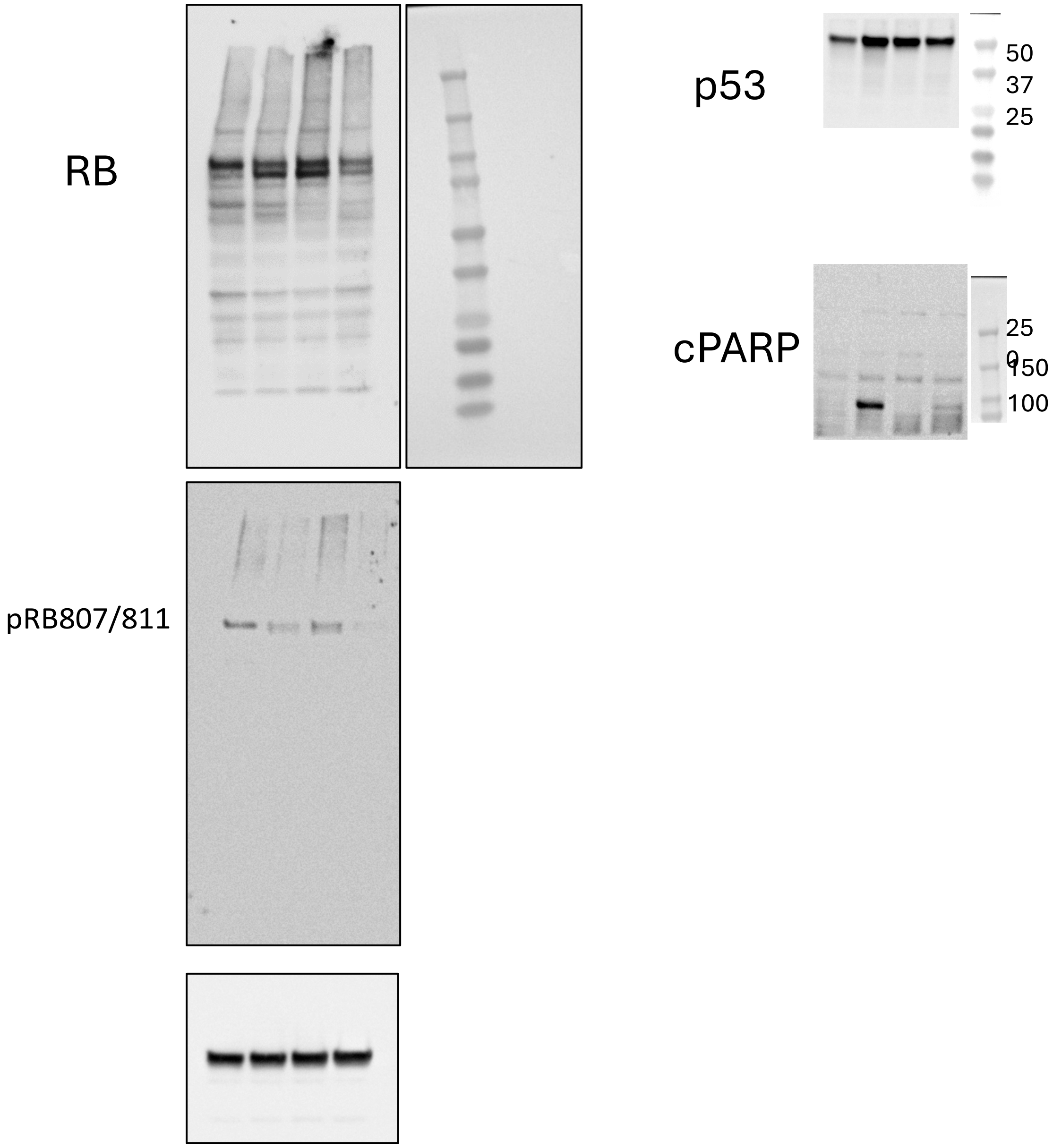

## Figure S5B

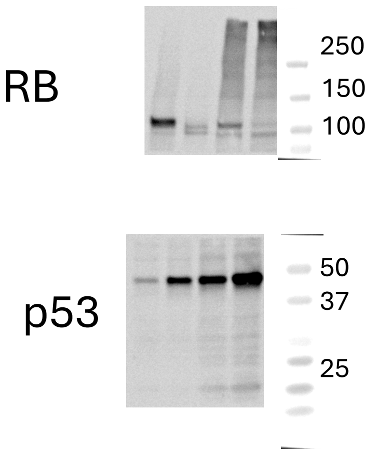


## Figure S5C

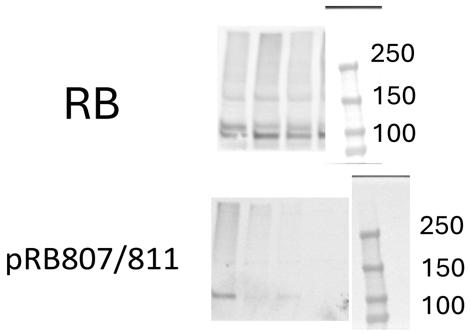

Supplement: Supplementary file 2 — Raw immunoblots [file 41419_2024_7204_MOESM2_ESM.pdf]
